# Sublaminar Band Fixation Provides Excellent Anchors for MAGEC Rod Distraction Systems

**DOI:** 10.5435/JAAOSGlobal-D-22-00164

**Published:** 2023-08-09

**Authors:** Samuel R. Rosenfeld, Matthew Weber, Evelyn S. Thomas, Kurt M. Barger

**Affiliations:** From Orthopaedic Surgery, Children's Hospital of Orange County (CHOC Children's Health), Orange, CA (Dr. Rosenfeld, Dr. Weber, Dr. Thomas, and Dr. Barger); Orthopaedic Surgery, Riverside University Health system Medical Center, Moreno Valley, CA (Dr. Weber and Dr. Barger); and Orthopaedic Surgery, Kettering Health Dayton, Dayton, OH (Dr. Thomas).

## Abstract

MAGEC rods (NuVasive) provide distraction growth in early-onset scoliosis. Pedicle screw use with MAGEC rods can lead to anchor failure. Sublaminar bands offer superior fixation points for the MAGEC system while preserving pedicles and facets, avoiding spinal cord injury, and eliminating the need for fluoroscopy. Sublaminar bands can be safely used up to cervical vertebra four (C4), substantially decreasing the risk of complications such as anchor pull-out, rod breakage, and proximal junctional kyphosis that typically occurs with pedicle screws and hooks. This case demonstrates the viable option of sublaminar band fixation as an anchor system for MAGEC rods. This is a retrospective case review of one patient with early-onset scoliosis who underwent multiple osteotomies, spinal cord decompression, and placement of MAGEC rods with sublaminar bands. The patient had successful distraction procedures conducted routinely throughout a 44-month period with no associated implant complications or neurologic sequelae during that period. The patient had achieved maximal distraction with the implanted rods and thereafter underwent removal of the MAGEC rods and replacement implantation with longer MAGEC rods. The purpose of this case review was to demonstrate the superior fixation results provided with sublaminar band fixation for MAGEC rod distraction systems.

## Background

MAGEC rods (NuVasive) have been shown to provide distraction growth in early-onset scoliosis (EOS).^1^ The major complications associated with the use of MAGEC rods have been anchor failure/pull-out, broken rods, growth mechanism failure, prominence, and infection.^2,3^ In one systemic analysis, the unplanned revision rate was as high as 33%.^4^ Rib anchors have been suggested as an improvement, but are not completely successful.^5^ Sublaminar band fixation offers “bicortical” lamina fixation without disrupting the pedicles or facet joints or concern for spinal cord injury and without the need of exposing the involved medical staff and patient to unnecessary radiation using fluoroscopic guidance. Sublaminar band fixation can also reduce the incidence of proximal junctional kyphosis that typically occurs with pedicle screws and hooks.^1^ Up to six levels of anchors, three on the proximal end and three on the distal end, can be used without arthrodesis of these normal spinal segments. We hypothesized that it is possible to reduce complications related to anchor failure in the MAGEC rod distraction system with the use of sublaminar band fixation.

## Case Presentation

**Figure 1:**
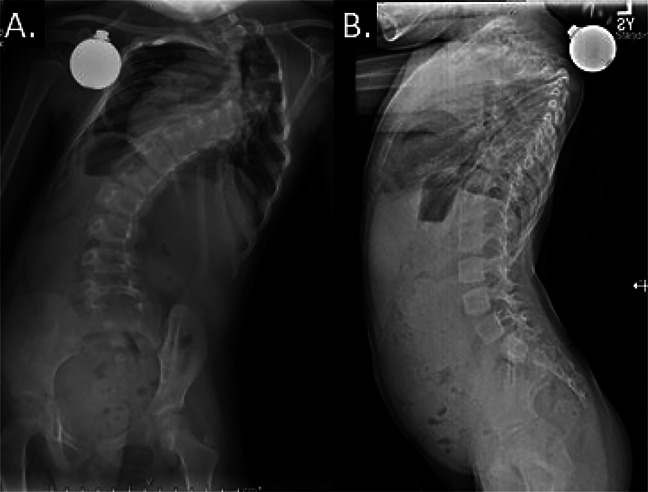
Preoperative radiographic images demonstrating early-onset scoliosis. **A**, PA view demonstrating the coronal scoliotic curve. **B**, Lateral view demonstrating the sagittal curve.

**Figure 2:**
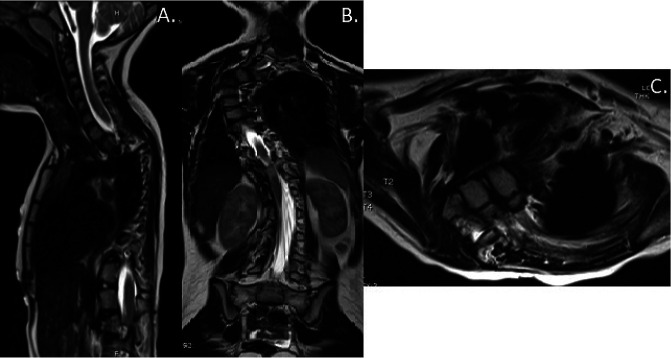
Radiographs showing preoperative magnetic resonance imaging of the patient's early-onset scoliosis. **A**, Sagittal cut of the cervical, thoracic, and lumbar spine. **B**, Coronal cut of the cervical, thoracic, and lumbar spine. **C**, Axial cut of T2, T3, and T4 vertebrae.

A four-and-a-half-year-old girl with a history of paraspinal embryonal rhabdomyosarcoma diagnosed at birth and status post tumor resection at 14 months of age presented to our clinic for management of early-onset thoracogenic scoliosis. The neoplasm had spanned from C6-L2 and involved the right erector spinae muscle, right posterior medial chest wall, right paramedian spine, and upper thoracic spinal canal, and the patient had reverse S-shaped thoracolumbar scoliosis. Extensive resection of the right erector spinae muscles was done at the time of tumor resection, along with resections of right T1-T9 rib heads, right T2-T6 posterior medial rib, right T1-T6 posterior medial intercostal muscles, right C7-T9 transverse process, right T1-T3 deep paraspinous mass, right T2-T3 neurovascular bundles, and right T2-T3 pedicles and decompression at right T2-T3, and a latissimus dorsi flap was used to fill the defect of the absent erector spinae musculature. She completed chemotherapy and was treated in a thoracic lumbar sacral orthosis because of her substantially weakened spine from the large resection that was done to help clear her tumor burden. Over the span of 3 years, the patient's spine progressed to a 77° dextroscoliotic curve in the T1-T10 region and a 39° levoscoliotic curve in the T10-L4 region. Throughout that time, she developed severe restrictive pulmonary disease because of her chest wall deformity and scoliosis. At 4 years of age, progressive myelopathy with bowel and bladder incontinence was noted. She developed lower extremity pain and weakness, which led to inability to independently ambulate. MRI with contrast showed no recurrent neoplasm but severe scoliosis and spinal stenosis at T3-T4. To alleviate her symptoms and EOS, we deemed her a candidate for decompression and osteotomies with MAGEC rod placement with sublaminar band fixation (Figures 1 and 2).

**Figure 3:**
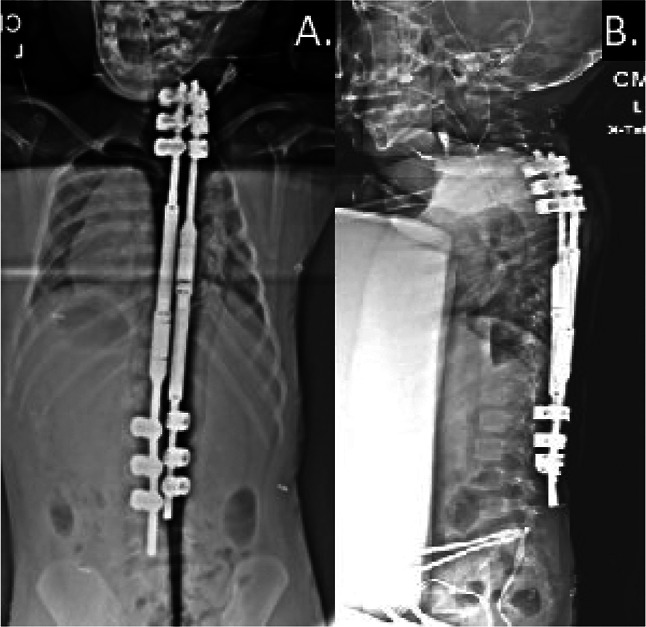
Postoperative radiographic images demonstrating MAGEC rods immediately after placement of the rods. **A**, PA view demonstrating coronal alignment of the cervical, thoracic, and lumbar spine. **B**, Lateral view demonstrating sagittal alignment of the cervical, thoracic, and lumbar spine.

An incision from C6-L2 was made over the scar from the prior neoplasm resection. The spinal cord was decompressed from T3-T4 with osteotomies. Additional osteotomies were done to further decompress the spinal cord because of no improvement in neuromonitoring signals. After decompression, the interspinous ligament and ligamentum flavum from C6-2 and T12-L2 were removed to facilitate the placement of the sublaminar bands from C7-T2 and T12-L2. The left side of the spine was instrumented first with a 5.5-mm distracting MAGEC rod cut to the appropriate length and contoured to facilitate normal sagittal alignment. The bands were then tightened with LigaPASS connectors (Medicrea). Similar corrective maneuvers were used on the right side with a 5.5-mm MAGEC reverse lengthening rod cut to the appropriate length and contoured to the appropriate sagittal alignment. The right MAGEC rod was connected to the spine with three superior and three inferior LigaPASS connectors from C7-L2 and sequentially tightened to correct the spinal deformity (Figure 3). The wound was irrigated, closed, and dressed appropriately.

**Figure 4:**
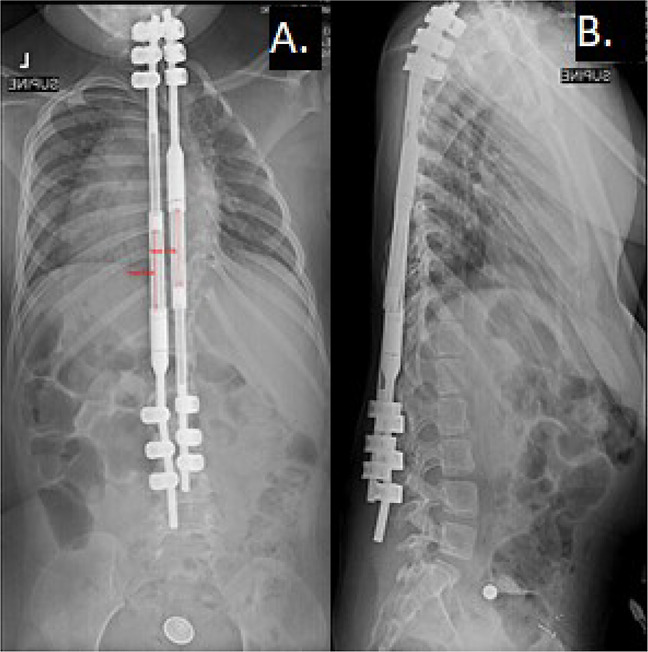
Four-year postoperative radiographic images demonstrating MAGEC rods at maximal distraction. The right rod was maximally distracted to 47.7 mm, and the left rod was maximally distracted to 48.3 mm. **A**, PA view. **B**, Lateral view.

**Figure 5:**
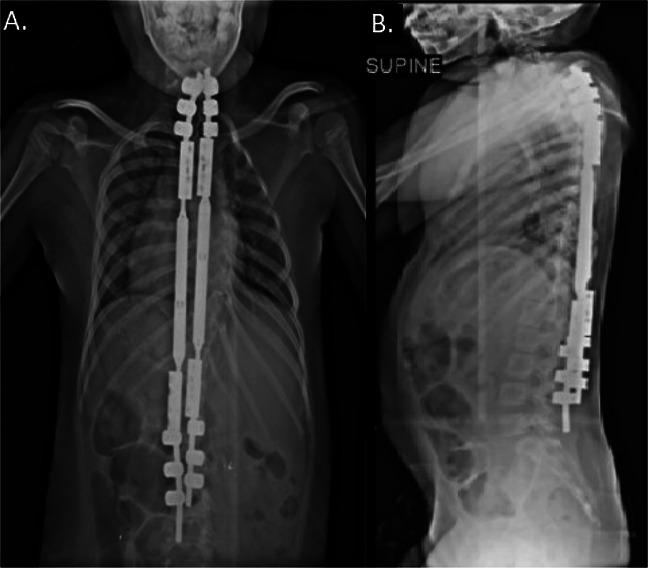
Radiographic images of the patient after MAGEC rod exchange. **A**, PA view. **B**, Lateral view.

The patient initially had weekly 1-mm MAGEC distractions for the first 4 weeks postoperatively, followed by monthly 1-mm MAGEC distractions for a total of 44 spinal lengthening procedures over the span of 44 months to achieve maximal distraction with the MAGEC rods. Maximal distraction was noted with a clicking sound on attempting additional distraction and confirmed on radiograph (Figure 4). No associated implant complications or neurologic sequelae were observed throughout this period. The patient underwent an additional procedure to exchange the MAGEC rods for a longer set of MAGEC rods 47 months after the initial implantation of the MAGEC rods because of the patient being skeletally immature and having many years of growth remaining. The initial implanted rods were cut proximally and distally, removed, and replaced with a new set of MAGEC rods, a left standard rod and a right reverse rod. End-to-end connectors were placed on the rods, and the anchors were unchanged. The correction of the spinal deformity was maintained, and the connectors were tightened (Figure 5). The wound was irrigated, closed, and dressed appropriately.

The initial implanted rods did not show any notable signs of wear or breakage. They had been able to maintain correction of the spinal deformity throughout the time the rods had remained in the patient. The patient is routinely followed, and distraction is done regularly with a goal of 1.2-centimeters distraction per year.

## Discussion

Although the MAGEC rod system is an approved method for correcting EOS, many studies have highlighted the high complication rates that can occur with this system. Many of the complications that occur are unplanned revision surgeries, anchor pull-out, implant failure, and rod breakage.^2-4^

A common reason for implant failure of MAGEC rods is failure of the distraction mechanism. This kind of failure may be due to accelerated distraction over a short period of time.^2^ As suggested by Agarwal et al, the patient's spine was distracted at a rate of 1 mm biweekly over the course of 4 weeks immediately after the first set of MAGEC rods were implanted and then at a rate of 1 mm per month. The patient did not have any complaints with this method of treatment, and no problems were noted on physical examination of the patient.^2^ The sagittal plane was also maintained after the exchange of MAGEC rods.

Sublaminar bands can be used to successfully anchor rods and correct the deformity of scoliotic spines, and even in neuromuscular scoliosis.^6^ Two or three-level sublaminar band fixation in the cervical, thoracic, and/or lumbar spine can be used as anchors with the MAGEC rod without disrupting the pedicle or facet joint and without arthrodesis, eliminating the need for pedicle screws, hooks, and/or rib anchors.^5^ These other methods of fixation of MAGEC rods to the vertebrae typically require two to three levels of fixation as well. We were able to use sublaminar band fixation safely up to the C4 level, avoiding proximal junctional kyphosis, a complication that can occur with the use of pedicle screws or hook and pedicle screw constructs.^4,7,8^ MAGEC rods are typically fixated in the upper thoracics, no further than T1 or T2 because of concerns of the occurrence of proximal junctional kyphosis and thus requiring correction later in life.^9^ Sublaminar bands provide “bicortical” fixation without arthrodesis at the anchor sites and are safer to place than pedicle screws or hooks in the immature, deformed spine. The bands are less prominent than rib hook fixation and can also be used for rib anchors. The sagittal plane of the spine can be addressed by placing the end-to-end connectors adjacent to the anchors or adjacent to the MAGEC rod. Sublaminar bands also do not require fluoroscopy during fixation, thus reducing radiation exposure. We demonstrate that sublaminar bands are a safe method of fixation and a viable option for successful MAGEC rod fixation in a young, skeletally immature patient.

In conclusion, we present a case of a young patient who had successful implantation and maintenance of MAGEC rods fixated with sublaminar bands for over 4 years, and this patient has been followed for 51 months to date, which is one of the longest average follow-ups reported in the literature for patients implanted with MAGEC rods for EOS.^3,10,11^ This case report is one of many examples that show the potential of sublaminar bands’ success in scoliosis correction and now with the use of MAGEC rods.
